# Characteristics of emergency general surgery services in Switzerland: a nationwide survey

**DOI:** 10.1007/s00068-023-02272-2

**Published:** 2023-07-20

**Authors:** Oliver Quaile, Stéphanie Fabienne Perrodin, Amedeo Trippel, Beat Schnüriger, “SwissACS”

**Affiliations:** 1 Department of Visceral Surgery and Medicine, Inselspital, Bern University Hospital, University of Bern, Bern, Switzerland; 2 Swiss Association for Trauma and Acute Care Surgery, Bern, Switzerland

**Keywords:** Emergency general surgery, Acute care surgery, Service models, Surgeons on-call

## Abstract

**Objective:**

Running an emergency general surgery (EGS) service is challenging and requires significant personnel and institutional resources. The aim of this study was to achieve a nationwide overview of the individual EGS service organizations in public hospitals in Switzerland.

**Methods:**

All Swiss public hospitals with a surgical and emergency department were included and contacted by telephone. General surgeons were interviewed between December 2021 and January 2022 using a standardized questionnaire.

**Results:**

Seventy-two out of 79 public hospitals in Switzerland (91.1%) agreed to the survey. They employed 1,581 surgeons in 19 (26.4%) hospitals with < 100 beds, 39 (54.2%) hospitals with 100–300 beds, 7 (9.7%) with 300–600 beds, and 7 (9.7%) with > 600 beds. The median number of surgeons per hospital was 20.5 (IQR 13.0–29.0). Higher level of care (intermediate or intensive care unit) was significantly less available in small hospitals (< 100 beds). The median hour of designated emergency operating room capacity per day was 14 h (IQR 14–24) for all hospitals with < 600 beds and 24 h (IQR 14–24) for the largest hospitals (> 600 beds). With increasing hospital size, there was a significant increase in the number of surgical units where EGS and orthopedic trauma surgery were covered by two separate teams (21.1% vs. 43.6% vs. 85.7% vs. 100%, *p* = 0.035)*.* The median number of surgeons on-call per hospital and per 24 h was 5.0 (IQR 3.3–6.0).

**Conclusion:**

Lack of higher level of care in small hospitals, limited emergency OR capacity and short rotations of on-call teams are major drawbacks of many current EGS systems in Switzerland. Centralization of critically ill EGS patients and reorganization of surgical on-call systems to designated acute care surgery teams should be considered.

**Supplementary Information:**

The online version contains supplementary material available at 10.1007/s00068-023-02272-2.

## Introduction

Running emergency general surgery (EGS) services is challenging and requires significant personnel and institutional resources [1–4]. In addition, increasing specialization of surgeons has made the establishment of EGS services more difficult [5–7].

In 2008, Uranues et al. performed a questionnaire-based survey including 18 European countries. These authors concluded that EGS was not recognized as a separate specialty and EGS interventions were mostly performed by surgical subspecialists according to anatomical region (abdominal, orthopedic trauma, thoracic, vascular) irrespective of whether it was a trauma or non-trauma patient [1]. In addition, at the time of this investigation, only few hospitals had designated operating room (OR) capacities for EGS.

The implementation of acute care surgery (ACS) in the United States 2003 [8] and Australia 2008 [5] replaced the traditional EGS on-call service model where EGS interventions are performed by rotating surgeons with different subspecializations and often during or after the elective operations. In contrast, the ACS model includes a designated surgical team providing a 24/7 care for all EGS and trauma patients including surgical critical care [9–12]. A designated ACS team reduces time to surgery, perioperative mortality, risk of postoperative complications, admission to intensive care units and the length of hospital stay in patients with acute surgical disease [13–15].

Currently, no detailed data are available on the EGS service models in Switzerland. The aim of this survey was to assess the current resources and characteristics of EGS services in public hospitals in Switzerland.

## Methods

All public hospitals with a surgical and emergency department (ED) listed by the Swiss Government and the regional (cantonal) offices of public health [16, 17] were contacted by telephone. The phone interviews comprised a standardized questionnaire including 17 questions covering clinical structure (total hospital beds, infrastructure [OR, ED, intensive care unit (ICU), intermediate care unit (IMC)], and the number of surgeons), organization of the surgical on-call service (number of surgeons on-call, duration of on-call rotation), and work characteristics of the surgical team on-call (see entire questionnaire in Appendix I). After oral consent, the interviews were conducted with a consultant or an attending surgeon between December 2021 and January 2022 by four interviewers (OQ, SFP, AT, and BS).

### Workforce analysis

The surgeons within each surgical unit were categorized as residents (non-board-certified surgeons in training), consultants (board-certified general surgeons), and attending surgeons (certified general surgeons in senior leading position). The calculated number of surgeons on-call within 24 h per month (‘on-call-surgeon-days per month’) was calculated as following:

$$\text{On{-}call{-}surgeon{-}days\,per\,month}\,=\,\text{Total\,number\,of\,surgeons\,on{-}call\,per\,24\,h} \times 30\,\text{days.}$$

For example, a surgical on-call service with two residents, one consultant and one attending surgeon on-call per 24 h, equals 120 on-call-surgeon-days per month (4 surgeons × 30 days).

In order to compare the personnel resources required to provide the surgical on-call service between the hospitals, the workforce ratio was calculated as following:

$$Workforce\,ratio\, = \,On {-} call {-} surgeon {-} days/Total\,surgeon {-} days.$$

For example, four surgeons on-call for 24 h in a surgical department with a total of 20 surgeons who could potentially be on-call, the workforce ratio equals 0.2 (4 on-call surgeons/20 employed surgeons).

### Cost analysis for surgeons on-call

The cost analysis for the annual EGS service was calculated as following: total number of surgeons on-call per 24 h multiplied by the annual salary. According to the Swiss Medical Association (FMH) [18], the mean annual salary for residents was set at 96,576 euros (EUR), 155,861 EUR for consultants and 280,168 EUR for attending surgeons. The monthly average exchange rate of May 2022 (1 EUR = 1.0458 Swiss francs) was used as a reference.

### Statistical analysis

Results are presented as median and interquartile ranges or percentages as appropriate. Differences between two groups were compared using the Fisher-exact Test for categorical data and Mann–Whitney *U* Test for continuous variables. For categorical variables, the Kruskal–Wallis Test or Chi-Square was applied to compare medians and proportions, respectively. The level of significance was set at *p* < 0.05. Statistical analysis was performed using SPSS statistical software version 28 (SPSS, Chicago, IL, USA).

## Results

Overall, 79 hospitals met the inclusion criteria and were contacted by the interviewers. Of those, 72 (91.1%) surgical departments participated in the telephone survey and were included in the analysis. These 72 surgical units were located in 19 (26.4%) hospitals with < 100 beds, 39 (54.2%) hospitals with 100–300 beds, 7 (9.7%) with 300–600 beds, and 7 (9.7%) with > 600 beds.

At the time of the interview, a total of 1,581 surgeons were employed in the 72 surveyed surgical departments including 833 (52.7%) residents, 376 (23.8%) consultants and 372 (23.5%) attending surgeons. The median number of surgeons per hospital was 20.5 (IQR 13.0–29.0). These included 11.0 (IQR 7.0–15.8) residents, 5.0 (IQR 2.3–7.0) consultants, and 5.0 (IQR 3.0–5.0) attendings. Table 1 shows the number of surgeons stratified according to the size of the hospital.

**Table 1:** Specific staff, infrastructural and emergency service characteristics by hospital size (number of beds)

	Total *n* = 72	< 100 *n* = 19	100–300 *n* = 39	300–600 *n* = 7	> 600 *n* = 7	*p*-value
**Staff**						
*Residents*	11.0* [IQR 7.0–15.8]	6.0* [IQR 5.0–7.0]	12.0* [IQR 8.0–16.0]	17.0* [IQR 11.0–19.0]	17.0* [IQR 15.0–21.0]	< 0.001
*Consultant surgeons*	5.0* [IQR 2.3–7.0]	2.0* [IQR 0.0–3.0]	5.0* [IQR 4.0–7.0]	7.0* [IQR 6.0–10.0]	12.0* [IQR 10.0–14.0]	< 0.001
*Attending surgeons*	5.0* [IQR 3.0–5.0]	3.0* [IQR 2.0–4.0]	5.0* [IQR 5.0–7.0]	5.0* [IQR 4.0–7.0]	8.0* [IQR 7.0–11.0]	< 0.001
*Total surgeons*	20.5* [IQR 13.0–29.0]	10.0* [IQR 8.0–13.0]	23.0* [IQR 17.0–29.0]	28.0* [IQR 21.0–36.0]	41.0* [IQR 33.0–42.0]	< 0.001
**Infrastructure**						
ED/ICU/IMC						< 0.001
*ED* + *ICU*	57 (79.2)	6 (31.6)	37 (94.9)	7 (100)	7 (100)	
*ED* + *IMC without ICU*	8 (11.1)	7 (36.8)	1 (2.6)	0	0	
*ED without IMC/ICU*	7 (9.7)	6 (31.6)	1 (2.6)	0	0	
Designated emergency OR capacity						
*Median hours of designated* *emergency OR capacity per day*	14.0* [IQR: 14–0-24.0]	14.0* [IQR: 14–0-14.0]	14.0* [IQR: 14–0-24.0]	14.0* [IQR: 14–0-24.0]	24.0* [IQR: 14–0-24.0]	0.007
*In/after elective program* + *night* *(5 p.m.–7 a.m.)*	45 (62.5)	13 (68.4)	25 (64.1)	5 (71.4)	2 (28.6)	0.041
*24 h*	20 (27.8)	2 (10.5)	11 (28.2)	2 (28.6)	5 (71.4)	
*In/after elective program* + *no OR* *at night*	5 (6.9)	4 (21.1)	1 (2.6)	0	0	
*12 a.m.–8 a.m*	2 (2.8)	0	2 (5.1)	0	0	
**Surgical on-call service characteristics**						
*24 h*	68 (94.4)	16 (84.2)	38 (97.4)	7 (100)	7 (100)	0.152
*Solely weekdays (7 a.m.–7 p.m.)*	4 (5.6)	3 (15.8)	1 (2,6)	0	0	
Organization ED						< 0.001
*24 h surgeon on-call, no ED* *physician*	41 (56.9)	18 (94.7)	19 (48.7)	4 (57.1)	0	
*24 h ED physicians*	17 (23.6)	0	8 (20.5)	2 (28.6)	7 (100)	
*Day ED physician/night* *surgeon on-call*	14 (19.4)	1 (5.3)	12 (30.8)	1 (14.3)	0	
Organization EGS and orthopedic, trauma surgery						0.035
*Two separate teams*	34 (47.3)	4 (21.1)	17 (43.6)	6 (85.7)	7 (100)	
*One team*	27 (37.5)	12 (63.2)	15 (38.5)	0	0	
*Depends on consultant/attending*	6 (8.3)	2 (10.5)	3 (7.7)	1 (14.3)	0	
*One team/two attendings*	5 (6.9)	1 (5.3)	4 (10.3)	0	0	

Values are numbers/median* (percentages)/[IQR: interquartile range] unless indicated otherwise. ED: Emergency department, IMC: Intermediate care unit, ICU: Intensive care unit, OR: Operation room, EGS: Emergency general surgery

The estimated proportion of EGS operations to the overall number of operations was 30–50% in 41 hospitals, 10–30% in 25 hospitals, < 10% in 4 hospitals, and 50–70% in 2 hospitals.

### Infrastructural characteristics

Table 1 shows specific infrastructural characteristics stratified according the size of the hospitals. All 72 hospitals have a 24/7 ED. Fifty-seven out of 72 hospitals (79.2%) have full capacity for higher level of care [intermediate care unit (IMC) and intensive care unit (ICU)]. Eight hospitals (11.1%) have an IMC but no ICU and seven hospitals (9.7%) have neither. Higher level of care (intermediate or intensive care) was significantly less available in small hospitals (< 100 beds) than in larger hospitals with > 100 beds (68.4% vs 97.5% for 100–300 beds vs. 100% for > 300 beds, *p* < 0.001).

Table 1 presents in detail the emergency OR capacity stratified according the hospital size. Of note, 20 hospitals (27.8%) have designated 24-h emergency OR availability. Forty-five hospitals (62.5%) have no designated emergency OR capacity at daytime (need for interruption of elective program for emergency cases between 7 a.m. and 5 p.m.) but a designated emergency OR at night (5 p.m.-7 a.m.).

The total median hour of designated emergency OR capacity per day was 14 h (IQR 14–14) for the smallest hospitals (< 100 beds), 14 h (IQR 14–24) for hospitals with 100–300 beds, 14 h (IQR 14–24) for hospital with 300–600 beds and 24 h (IQR 14–24) for the largest hospitals (> 600 beds) (Table 1).

**Fig. 1:**
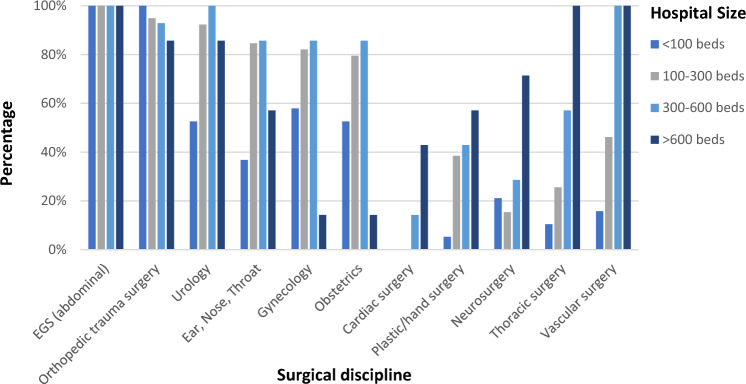
Interdisciplinary use of emergency operating room capacity stratified according the hospital size

Figure 1 exposes the interdisciplinary use of emergency OR capacity. In 94.4% of all surveyed hospitals, emergency OR capacities are shared between EGS and orthopedic trauma surgery [100% (< 100 beds) vs. 94.9% (100–300 beds) vs. 92.9% (300–600 beds) vs. 85.7% (> 600 beds)]. With increasing hospital size, other surgical subspecialties such as vascular surgery, cardiac surgery, thoracic surgery or neurosurgery are sharing the emergency OR availability. With decreasing hospital size gynecology and obstetrics as well as ear, nose and throat (ENT) are sharing the emergency OR availability with all other surgical disciplines.

### Surgical on-call service characteristics

Table 1 shows specific surgical on-call service characteristics stratified according the size of the hospitals. Sixty-eight (94.4%) of the 72 surveyed surgical units offer a 24/7 EGS service. Regarding the separation of the EGS and orthopedic trauma service, 34 hospitals (47.3%) have 2 separate teams. Twenty-seven (37.5%) have 1 team doing both EGS and orthopedic trauma service. Five hospitals (6.9%) have one on-call surgical resident and consultant, but 2 attending surgeons responsible for either EGS or orthopedic trauma. Six hospitals (8.3%) have one surgical on-call team (resident and consultant), however, have either one attending surgeon for both, EGS and orthopedic trauma surgery or a system of two separate attending surgeons for EGS or orthopedic trauma surgery.

With increasing hospital size, there is a significant increase in the number of surgical units with two separate on-call teams doing EGS or orthopedic trauma surgery (21.1% vs. 43.6% vs. 85.7% vs. 100%, *p* = 0.035) (Table 1).

### Responsibilities of the surgical on-call team

**Fig. 2:**
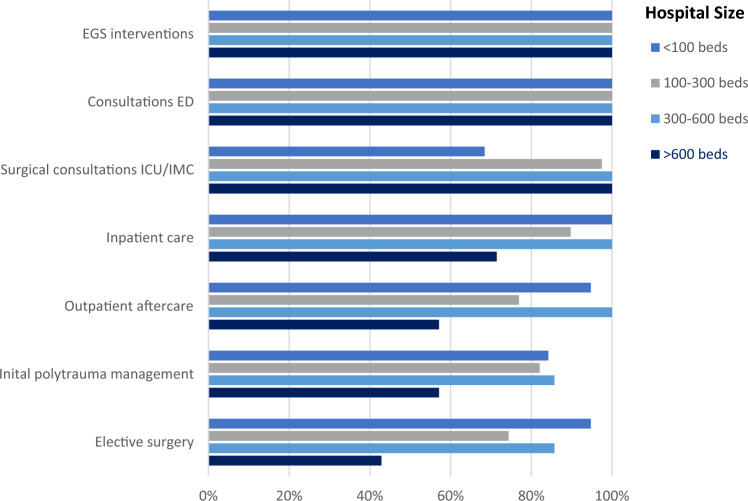
Specific responsibilities of the surgical on-call team

In all the 72 surgical units surveyed, the surgical on-call team carries out consultations in the ED and regular wards and performs EGS interventions. Moreover, in all the 57 hospitals with and IMC or ICU, the surgical on-call team carries out consultation on the IMC or ICU. In addition, the surgical on-call team is responsible for the initial polytrauma management in 54 of 65 (83.1%) hospitals with < 600 beds. This percentage decreases to 57.1% (4 of 7) of large hospitals with > 600 beds, where ED physicians are doing the initial evaluation of polytraumatized patients. In 77.8% (56 of 72) of the surveyed surgical departments, the on-call team also performs elective surgery during the on-call service. This proportion decreases with increasing size of hospital (94.3% vs. 74.4% vs. 85.7% vs 42.9%, *p* = 0.035) (Fig. 2).

### Workforce analysis and on-call service rotation system

Overall, the median number of surgeons on-call per hospital in 24 h was 5.0 (IQR 3.3–6.0). This results in a median on-call-surgeon-day per month per hospital of 150.0 (IQR 98.1–180.0). The on-call-surgeon-days per month was 75.0 (IQR 60.0–90.0) for residents, 30.0 (IQR 30.0–30.0) for consultants, and 30.0 (IQR 30.0–60.0) for attending surgeons, respectively.

The median workforce ratio per month of all surgeons on-call per hospital was 0.22 (IQR 0.18–0.29). This decreases with increasing size of hospital (Table 2*).*

**Table 2:** Workforce analysis of surgeons per hospital by hospital size (number of beds)

	Total *n* = 72	< 100 *n* = 19	100–300 *n* = 39	300–600 *n* = 7	> 600 *n* = 7	*p*-value
Median on-call-surgeon-days per month						
*Residents*	75.0 (IQR 60.0–90.0)	60.0 (IQR 30.0–60.0)	90.0 (IQR 60.0–90.0)	90.0 (IQR 90.0–90.0)	90.0 (IQR 60.0–90.0)	< 0.001
*Consultant surgeons*	30.0 (IQR 30.0–60.0)	24.0 (IQR 8.9–30.0)	30.0 (IQR 30.0–60.0)	30.0 (IQR 30.0–60.0)	30.0 (IQR 30.0–60.0)	0.009
*Attending surgeons*	30.0 (IQR 30.0–30.0)	30.0 (IQR 30.0–30.0)	30.0 (IQR 30.0–30.0)	30.0 (IQR 30.0–30.0)	30.0 (IQR 30.0–30.0)	1.000
*Total*	150.0 (IQR 98.1.-180.0)	90.0 (IQR 68.6–120.0)	150.0 (IQR 120.0–180.0)	150.0 (IQR 150.0–180.0)	174.0 (IQR 150.0–180.0)	< 0.001
Median workforce ratio per month						
*Residents*	0.20 (IQR 0.17–0.27)	0.24 (IQR 0.20–0.29)	0.20 (IQR 0.17–0.25)	0.18 (IQR 0.16–0.25)	0.18 (IQR 0.10–0.20)	0.017
*Consultant surgeons*	0.20 (IQR 0.17–0.33)	0.29 (IQR 0.17–0.50)	0.25 (IQR 0.20–0.33)	0.17 (IQR 0.14–0.20)	0.14 (IQR 0.08–0.15)	0.002
*Attending surgeons*	0.20 (IQR 0.17–0.33)	0.33 (IQR 0.21–0.50)	0.20 (IQR 0.15–0.31)	0.20 (IQR 0.20–0.25)	0.14 (IQR 0.11–0.17)	< 0.001
*Total*	0.22 (IQR 0.18–0.29)	0.30 (IQR 0.21–0.33)	0.22 (IQR 0.19–0.24)	0.18 (IQR 0.17–0.19)	0.15 (IQR 0.15–0.16)	< 0.001

Values are median (interquartile range) unless indicated otherwise

The number of in-house consultants at night increases with the size of the hospital [15.9% (< 100 beds) vs. 28.2% (100–300 beds) vs. 71.4% (300–600 beds)]. However, in the largest hospitals (> 600 beds), the majority of surgical consultants (57.1%) are on-call but not in-house at night. In 93.1% of all surveyed surgical departments, residents were in-house at night [94.7% (< 100 beds) vs. 89.8% (100–300 beds) vs. 100% (300–600 beds) vs. 100% (> 600 beds)].

In 19 of 72 surgical units (26.4%), residents are directly supervised by attending surgeons without a consultant. This model becomes less frequent as hospital size increases [73.7% (< 100 beds) vs. 26.3% (100–300 beds) vs. 0% (300–600 beds) vs. 0%, *p* < 0.001(> 600 beds)].

Table 3 summarizes duration of the EGS service rotation per surgeons according to hospital size. Overall, 54% of residents, 84% of consultants, and 95% of attending surgeons doing EGS switch on a daily base. With increasing hospital size, the duration of on-call rotations (typically one week) increases for residents and consultants. The proportion of hospitals with a rotation system of at least 1 month is 18.1% for residents, 4.8% for consultants and 0% for attendings.

**Table 3:** Duration of rotation system in the EGS service for surgeons per hospital size (number of beds)

	Total *n* = 72	< 100 *n* = 19	100–300 *n* = 39	300–600 *n* = 7	> 600 *n* = 7	*p*-value
Residents						0.01
*One day*	39 (54.2)	13 (68.4)	21 (53.8)	3 (42.9)	2 (28.6)	
*One week*	20 (27.8)	4 (21.1)	10 (25.6)	4 (57.1)	2 (28.6)	
*One month*	2 (2.8)	1 (5.3)	1 (2.6)	0	0	
*2–3 months*	9 (12.5)	1 (5.3)	7 (17.9)	0	1 (14.3)	
*4–6 months*	2 (2.8)	0	0	0	2 (28.6)	
> *6 months*	0	0	0	0	0	
Consultants*						0.004
*One day*	51 (82.3)	12 (100)	29 (80.6)	7 (100)	3 (42.8)	
*One week*	8 (12.9)	0	7 (19.4)	0	1 (14.3)	
*One month*	0	0	0	0	0	
*2–3 months*	1 (1.6)	0	0	0	1 (14.3)	
*4–6 months*	1 (1.6)	0	0	0	1 (14.3)	
> *6 months*	1 (1.6)	0	0	0	1 (14.3)	
Attendings						0.009
*One day*	68 (94.4)	19 (100)	37 (94.9)	6 (85.7)	6 (85.7)	
*One week*	4 (5.5)	0	2 (5.1)	1 (14.3)	1 (14.3)	

Values are numbers (percentage)

*Calculation included 62 of 72 surgical units. (10 surgical units do not have consultants in the EGS service)

### Cost analysis for surgeons on-call

The annual total costs for all surgeons on-call was 49,227,746 EUR. Median annual cost per hospital was 742,230 EUR (IQR 532,607–881,622). There were significant lower median costs for the surgeons on-call in the smallest hospital (< 100 beds) compared to hospitals with 100–300, 300–600 and > 600 beds [528,941 EUR (IQR 439,089–699,321) vs. 725,760 EUR (IQR 629,183–725,760) vs. 725,760 EUR (IQR 725,760–881,622) vs. 725,760 EUR (IQR 725,760–940,906), p < 0.001].

## Discussion

This is the first nationwide survey assessing the characteristics of EGS services in Switzerland. With inclusion of 72 of the 79 (91.1%) public hospitals including a 24/7 ED and surgical unit, a representative sample could be achieved. We found that every fifth surgeon in Switzerland is on-call (median workforce ratio of 0.22). The responsibilities of the surgeons on-call are numerous and highly relevant to the hospital’s resources and structure including daily IMC and ICU rounds, initial evaluation and triage of acute surgical patients (trauma and non-trauma) and performing EGS operations at day and night. In addition, the survey has identified important drawbacks and room for improvement in EGS systems in Switzerland. The most important are the high diversity of EGS on-call characteristics, a lack higher level of care, limited designated emergency OR capacities and the short duration of EGS on-call rotation.

The median number of surgeons on-call per 24 h per hospital was 5.0 (IQR 3.3–6.0). These included two to three residents, one to two consultants and one attending surgeon and is compatible with Swiss laws for working hour regulation [19]. The median workforce ratio was 0.22 (IQR 0.18–0.29). In other words, at all times, every fifth surgeon is involved in the EGS service, which underlines the personnel importance and impact on the on-call schedule. This value decreases with increasing size of surveyed hospital (0.30 to 0.15, Table 2) which emphasizes the significance of EGS in smaller hospitals.

The responsibilities of the surgical on-call team are multiple and highly relevant to the overall hospital system [20]. Besides the surgical skills in emergency and elective cases, knowledge regarding initial evaluation of acute surgical patients, ICU relevant topics and non-operative treatment strategies are required to overcome this unique role of in-house troubleshooting. Similarly to the US, the EGS teams in Switzerland are closely involved in the ICU management of critically ill surgical patients including polytraumatized patients. However, in Switzerland, the general surgical curriculum includes only 3 months of mandatory ICU training to acquire ICU-specific knowledge. We suggest that further compulsory critical care-specific training should be implemented early, especially for those surgeons involved in EGS.

Interestingly, in 77.8% of the surveyed surgical departments, the on-call team also performs elective surgery during the on-call service. This is important in order to maintain the surgical volume and training through elective surgery [21].

Several important drawbacks of EGS systems in Switzerland have been identified. Of note, every fifth hospital with a 24/7 ED and offering EGS does not have an ICU and 70% do not have designated emergency OR capacities at daytime. Moreover, the available OR resources are shared with other operative specialties including obstetrics or ENT. This finding is of concern, since several studies showed that higher level of care and designated daytime emergency OR capacities reduce morbidity in EGS patients [5, 22, 23]. Moreover, missing daytime OR capacities for EGS patients leads to a delay to potentially life-saving surgery with increasing risk of postoperative complications, longer hospital length of stay, increased admission to ICU and perioperative mortality [14, 24]. Stupart et al. investigated the impact of the implementation of a dedicated daily emergency OR managed by a designated on-site consultant by comparing 966 EGS patients before to 984 EGS patients after the implementation of a designated emergency OR. These investigators found that implementation of designated emergency OR capacities is associated with a shift from night- to daytime emergency surgery and subsequently to improved surgeons’ satisfaction [15]. This might be an explanation for one interesting finding of the current study, that in hospitals with designated daytime emergency OR capacities consultants are on-call (not necessarily in-house) during the night because the EGS cases may have been finished by the end of the day.

One important finding of the current survey was the overall very low continuity of EGS teams. Of note, 82% of residents, 95% of consultants and 100% of attending surgeons are rotating on a daily or weekly manner between EGS and elective surgical teams. These short-term rotations of EGS teams at all surgical levels results in an increased lack of continuity of care of EGS patients, where physiological and clinically relevant deterioration may occur and need to be identified in a timely manner.

Moreover, short-term rotations are associated with an increased number of handovers that are time consuming and a source of potential communication errors or loss of important information. Ye et al. showed that 15% of handovers were incomplete and missing important information [25].

Interestingly, the current investigation showed that longer EGS rotations were more frequent for residents and consultants in the largest hospitals compared to smaller hospitals (Table 3). The awareness and acknowledgment of the special needs and time sensitiveness of EGS patients leads to the implementation of designated acute care surgical teams aiming for increased continuity of care. Moreover, it has been showed that trauma and critical care trained surgeons provide more efficient care to patients suffering from EGS conditions compared to boarded-certified general surgeons with or without subspeciality titles (e.g., less time from ED to the OR) [26].

When EGS is provided by a hospital, a 24/7 ED, higher level of care, emergency OR capacity and continuity and formation of designated ACS teams including EGS and ICU care are critical and need to be emphasized and implemented with high priority. All these critical issues have a direct impact on effectiveness and outcome of acute care. Moreover, after the establishment of an ACS service, 38% increase in EGS cases was reported and as an added benefit, elective surgery admissions also increased by 30%. The implementation of an ACS service seemed to be a win–win solution: more surgical cases for the acute care surgeons and increased volume for the elective surgeons [21].

In the current study, less than 30% of the surveyed hospitals meet what we consider to be the four most important criteria for high-quality surgical acute care [24/7 designated and specifically trained ACS team, 24/7 available higher level of care (IMC or ICU), 24/7 ED, and 24/7 designated emergency OR capacity]. Around 50% of the hospitals meet only two of the four criteria and every fifth hospital meet solely one criteria. Possible reasons may be of organizational or financial character. Nevertheless, the transition to a designated ACS service is feasible without additional employment, however, does mandate reorganization of the staff and the recognition of the special needs of surgically acute sick patients. Moreover, similar to trauma systems, hospital networks need to be formed in order to further consolidate resources and with it, improving the outcomes of non-trauma EGS patients. In addition, the training of young surgeons towards ACS including ICU training should be optimized. Moreover, from the political standpoint, awareness of ACS being a surgical subspeciality taking into account the special requirements of surgically acute critically ill patients needs to be further promulgated.

### Limitations

Surgical skills or ACS experience and levels of knowledge of the residents, consultants or attending surgeons on-call have not been assessed in detail by the telephone interview. Moreover, a very short on-call rotation system does not necessarily mean low continuity. Especially in smaller hospitals with a small total number of surgeons and a daily on-call rotation system, the same team may manage patients in the further follow-up. Therefore, estimation of the quality of acute care of individual on-call systems is not feasible. Moreover, the cost analysis of EGS systems needs careful interpretation since the revenues were not available. Here, further studies on the cost effectiveness of a designated ACS service are warranted.

## Conclusions

This is the first nationwide survey on current organizational characteristics of EGS services in Switzerland. However, we believe that these findings here may be translated to other Western European Countries. A high diversity of EGS on-call service characteristics, lack of higher level of care, limited designated emergency OR resources and a lack of continuity of EGS teams were major drawbacks identified. This stands in contrast to the numerous, important and highly relevant responsibilities of the surgical on-call teams. These responsibilities are very relevant to the patients’ outcomes and overall success of the surgical unit. Further awareness and acknowledgment of the special needs and time sensitiveness of EGS patients is required. Implementation of designated ACS teams needs to be evaluated since it has been shown to be beneficial on multiple outcome levels.

### Supplementary Information

Below is the link to the electronic supplementary material.

Supplementary file1 (DOCX 12 kb)

## Data Availability

The datasets used and analyzed during the current study are available from the corresponding author on reasonable request.

## Appendix I—Questionnaires of the telephone survey

1. This survey is specifically addressed to surgical clinics and hospitals as well as hospitals that fulfil a center or primary care medical treatment contract (according to FOPH hospital types level 1–5). Does your hospital have a center or primary care medical treatment contract?

- Yes

- No (this concludes the survey)

2. In which major Swiss region is your hospital located?

- Région lémanique (VD, VS, GE)

- Espace Mittelland (BE, FR, SO, NE)

- Nordwestschweiz (BS, BL, AG)

- Zürich (ZH)

- Ostschweiz (GL, SH, AR, AI, SG, GR; TG)

- Zentralschweiz (LU, UR, SZ, OW, NW, ZG)

- Tessin (TI)

3. How many inpatient beds does your hospital have?

- < 100

- 100–200

- 200–300

- 300–400

- 400–600

- > 600

4. How many resident, consultant and attending surgeons does your surgical department have?

5. What is the percentage of emergency general surgery operations in your hospital?

- < 10%

- 10–30%

- 30–50%

- 50–70%

- > 70%

6. Your hospital has (multiple answers possible):

- Emergency department (24 h)

- Intensive care unit (24 h)

- Emergency department (daytime)

- Emergency department only daytime and weekdays (no emergency department at weekends)

- Intensive care unit only weekdays (no intensive care unit at weekends)

- Other (please specify)

7. Can emergency general surgery (e.g., appendectomy, cholecystectomy, incarcerated abdominal wall hernia, colon resection for perforation, etc.) be performed in your hospital at any time (24/7)?

- Yes

- No (please specify)

- Partly (please specify)

8. What surgical capacities are available in your hospital for emergency general surgery?

- We have separate emergency surgery capacity at all times (24/7) regardless of the elective surgery program

- We do not have separate operating capacities during the day and emergency operations are scheduled at short notice in the elective program. At night we have an emergency operation room at our disposal

- We do not have separate surgical capacities and emergency operations are scheduled at short notice in the elective program. In addition, we do not have any surgical capacity available at night

- Other system (please specify)

9. Often, surgical capacities for emergencies are shared with several surgical subspecialities in one operating theatre. With which subspecialities do you share these capacities in your hospital?

- Visceral surgery

- Traumatology

- Orthopedics

- Vascular surgery

- Cardiac surgery

- Thoracic surgery

- Plastic surgery

- Gynecology

- Obstetrics

- Urology

- ENT

- Neurosurgery

- Other (please specify)

10. Who is responsible for prioritizing emergency surgery in your hospital?

- Surgeon on call

- Anesthesiologist on call

- OR management

- Other (please specify)

11. Do you have an emergency general surgery service team 24/7 available in your hospital?

- Yes

- Partly (please specify)

- No (please specify)

12. Does your emergency general surgery service team care for both abdominal and orthopedic trauma emergencies?

- Yes

- No, we have two teams

- No, we have a different team division

13. What are the tasks/functions of your general emergency surgery service team?

- First contact/care on the emergency department

- Polytrauma management in the resuscitation room

- Assessment after initial contact (e.g., by emergency physicians)

- Surgical assessment at other clinics (e.g., internal medicine, orthopedics)

- Performing emergency general surgery operations

- Inpatient care of conservatively treated emergency patients (e.g., ileus, diverticulitis, splenic rupture)

- Co-supervision/surgical assessment in the intensive care unit

- Postoperative inpatient and outpatient follow-up care

- Performing elective general surgical operations

- Other (please specify)

14. Do residents and consultants rotate in the general emergency surgery services in your clinic for a defined period of time?

- Yes

- No, they all perform services in daily rotation

- Other system (please specify)

15. If yes: How long (weeks) and how many residents as well as consultants are totally rotated into the on-call service?

- Residents (numbers, rotation period)

- Consultants (numbers, rotation period)

16. Who is the primary supervisor for the residents and consultants on-call and how is this organized?

- All senior consultants/attendings on rotating background service

- Specific senior consultants/attendings on rotating background service

- Other system (please specify)

17. How many attendings share the on-call service?

## Declarations

### Congress presentation

Parts of the results were presented at the 11th Swiss Trauma & Acute Care Surgery Days (STACS Days), March 10–11, 2022, Bern, Switzerland and at the 109th Annual Congress of the Swiss Society for Surgery, June 2–3, 2022, Bern, Switzerland.
