# Supplementary material for: Characteristics of emergency general surgery services in Switzerland: a nationwide survey
Source: Eur J Trauma Emerg Surg. 2023 Jul 20;50(1):259–68. doi: 10.1007/s00068-023-02272-2 (PMC10923733; doi:10.1007/s00068-023-02272-2)
Supplement: Supplementary file 1 — Supplementary file1 (DOCX 12 kb) [file 68_2023_2272_MOESM1_ESM.docx]

**Supplemental material**

**Questionnaires of the telephone survey**

1. This survey is specifically addressed to surgical clinics and hospitals as well as hospitals that fulfil a center or primary care medical treatment contract. Does your hospital have a center or primary care medical treatment contract?

2. In which major Swiss region is your hospital located?

3. How many inpatient beds does your hospital have?

4. How many resident, consultant and attending surgeons does your surgical department have?

5. What is the percentage of emergency general surgery operations in your hospital?

6. Your hospital has (multiple answers possible):

- Emergency department (24hrs)

- Intensive care unit (24hrs)

- Emergency department (daytime)

- Emergency department only daytime and weekdays (no emergency department at weekends)

- Intensive care unit only weekdays (no intensive care unit at weekends)

- Other (please specify)

7. Can emergency general surgery (e.g., appendectomy, cholecystectomy, incarcerated abdominal wall hernia, colon resection for perforation, etc.) be performed in your hospital at any time (24/7)?

8. What surgical capacities (operating room availability) are available in your hospital for emergency general surgery?

9. Often, surgical capacities for emergencies are shared with several surgical subspecialities in one operating theatre. With which subspecialities do you share these capacities in your hospital?

10. Who is responsible for prioritizing emergency surgery in your hospital?

11. Do you have an emergency general surgery service team 24/7 available in your hospital?

12. Does your emergency general surgery service team care for both abdominal and orthopedic trauma emergencies?

13. What are the tasks of your general emergency surgery service team?

14. Do residents and consultants rotate in the general emergency surgery services in your clinic for a defined period of time?

15. If yes: How long (weeks) and how many residents as well as consultants are totally rotated into the on-call service?

16. Who is the primary supervisor for the residents and consultants on-call and how is this organized?

17. How many attendings share the on-call service?
